# Competences to self-manage low back pain among care-seeking adolescents from general practice - a qualitative study

**DOI:** 10.1186/s12875-023-02212-4

**Published:** 2023-11-29

**Authors:** Christian Lund Straszek, Lotte Stausgaard Skrubbeltrang, Kieran O’Sullivan, Janus Laust Thomsen, Michael Skovdal Rathleff

**Affiliations:** 1 Center for General Practice at Aalborg University, Aalborg, Denmark; 2 Department of Health Science and Technology, Aalborg University, Aalborg, Denmark; 3 Department of Physiotherapy, University of Northern Denmark, Aalborg, Denmark; 4 School of Allied Health, Health Research Institute, University of Limerick, Limerick, Ireland; 5 Ageing Research Centre, Health Research Institute, University of Limerick, Limerick, Ireland; 6 Sports and Human Performance Research Centre, University of Limerick, Limerick, Ireland

**Keywords:** Adolescents, General practice, Primary care, Low back pain, Function

## Abstract

**Background:**

There is limited knowledge about when and how adolescents with low back pain (LBP) interact with health care providers. This limits our understanding of how to best help these young patients. This study aimed to understand when and how care-seeking adolescents with LBP interact with health care providers and which health literacy competencies and strategies do they use to self-managing their LBP.

**Method:**

Ten semi-structured interviews (duration 20–40 min) were conducted online among adolescents aged 15–18 with current or recent LBP (pain duration range; 9 months – 5 years). The interview guide was informed by literature on health literacy and self-management in patients. We conducted a semantic and latent thematic data analyses.

**Results:**

Three major themes emerged from the analysis: (1) Self-management, (2) Pain and Function, and (3) Communication. All adolescents were functionally limited by their pain but the main reason to consult a health care provider was an increase in pain intensity. Many were able to navigate the healthcare system, but experienced difficulties in communicating with health care providers, and many felt that they were not being taken seriously. Their first line self-management option was often over-the-counter pain medicine with limited effects. Most adolescents expressed a desire to self-manage their LBP but needed more guidance from health care providers.

**Conclusion:**

Adolescents with LBP seek care when pain intensifies, but they lack self-management strategies. Many adolescents want to self-manage their LBP with guidance from health care providers, but insufficient communication is a barrier for collaboration on self-management.

**Supplementary Information:**

The online version contains supplementary material available at 10.1186/s12875-023-02212-4.

## Background

Low back pain (LBP) is one of the most common pain complaints in adolescence with a mean lifetime prevalence of 39% [1]. LBP is the most common reason for adolescents with musculoskeletal pain to consult their general practitioner with a consultation rate of 228 per 10.000 persons [2, 3]. Adolescent LBP is associated with high pain levels, worries and impaired function [2, 4]. Although a high lifetime prevalence of LBP in adolescent school children has been well established [1] less focus had been directed towards care-seeking adolescents with LBP.

Previous research has cross-sectionally investigated baseline demographics in care-seeking adolescents from general practice [2]. Care-seeking behavior among adolescents from general practice was in addition assessed in a register-based study [3]. Although these findings indicate that these adolescents display high LBP intensity, functional limitations, and worries [2], little is known as to when and for what specific reason adolescents with LBP decide to consult their general practitioner. This aspect is important to uncover, as care-seeking adolescents with musculoskeletal pain differ from non-care-seeking adolescents with such pain complaints [5].

More than 35% of adolescents continue to experience LBP into adulthood [6] with some experiencing LBP for more than 6 years after pain onset [4]. LBP is the highest-ranking musculoskeletal pain complaint among adults in terms of disability-adjusted life years [7]. Previous qualitative studies demonstrate that adults with LBP worry substantially about how their pain may influence their work capacity, social lives, and ability to get healthcare in the future [8, 9]. This underlines the need for a deeper understanding of how care-seeking adolescents manage their LBP and how they navigate the healthcare system and engage with health care providers. For this endeavor, one needs to be familiar with the terms of health literacy and self-management.

Health literacy has previously been described as an individual’s capacity to engage with the complex demands regarding health in the modern society [10]. As such, having adequate health literacy competencies means that and individual can take responsibility for one’s own health by obtain, process, appraise, and apply health information to promote and maintain good health [11]. Self-management is closely linked to health literacy and describes how an individual (with or without pain or disease) manages their own health in their everyday lives [12]. As described by Lorig and colleges, self-management is composed of five core-skills: problem solving, decision making, resource utilization, forming of a patient/health care provider partnership, and taking action [12].

Despite the vast importance of these terms in managing one’s health, little is known regarding health literacy competencies and self-managing strategies among care-seeking adolescents with LBP. Therefore, we aimed to conduct the current interview-based study among care-seeking adolescents with current or prior LBP to assess their health literacy competencies and self-management strategies to answer the following research questions.

### Research question

When and how do care-seeking adolescents with LBP interact with health care providers and which health literacy competencies and strategies do they use to self-managing their LBP?

## Methods

### Ethical considerations

All adolescents in the study received oral and written information about the purpose of the study prior to participating. Written informed consent was obtained from all adolescents prior to undertaking the interview. For interview studies among adolescents from Denmark between 15 and 18 years of age, there is no requirement for obtaining written informed consent from parents/legal guardians prior to undertaking the study. However, researchers are required to inform parents/legal guardians about the project in which the adolescents participate. Therefore, for all participants under the age of 18, parents/legal guardians were forwarded written information about the study. These guidelines are outlined by the Danish Data Protection Agency and the approach was further checked and confirmed by the local Ethics Committee. In Denmark, interview studies are exempt from obtaining approval from an ethics committee as this type of research does not fall within the legal boundaries of the regional ethics committees (LBK nr 1338 of 01/09/2020). Nevertheless, the Ethics Committee in the Northern Region of Denmark (Niels Bohrs Vej 30, 9220 Aalborg East, Denmark. The Region of Northern Denmark) was informed about the study approach outlined above and replied that no ethical approval was required (journal number: 2023 − 000206). The study was conducted in accordance with the Declaration of Helsinki.

### Recruitment

The current study was conducted at the Center for General Practice at Aalborg University, Denmark. Participants were recruited through primary care physiotherapy and medical clinics, sports clubs, and social media. Eligible adolescents had to meet the following criteria to be included in the study:

- Aged between 15 and 19 years.
- Experiencing constant or fluctuating activity-limiting LBP within the previous 12 months (i.e., the pain had to limit one or more of the adolescent’s daily activities).
- Previous contact with a health care provider due to LBP (e.g., general practitioner, physiotherapist, chiropractor) within the previous 12 months.

All potential adolescent informants were screened over the phone for eligibility by the first author. This was done to ensure that the included informants fulfilled the eligibility criteria prior to enrolment in the study.

### Research team and reflexivity

The first author, CLS, conducted all interviews. CLS is a trained physiotherapist with 2 years of clinical experience managing musculoskeletal pain complaints in both younger and older patients referred to rehabilitation in the municipality. CLS is currently a Ph.D. student at Aalborg University. At the time of the study, CLS had some prior experience with interview-based research although the current study was his first major project within the qualitative research paradigm. CLS was supervised throughout the study by several experienced researcher with extensive knowledge regarding semi-structured interviews and thematic data analysis. There was neither a personal nor a professional relationship between CLS and the adolescent participants prior to undertaking the interviews. The adolescents were informed about the objective of the study prior to enrolment. They were in addition informed about the interviewer’s background and special interest in the topic.

To heighten transparency, the authors want to state that they work under the assumption that an intervention for adolescent LBP to some extent should include components to develop sufficient self-management strategies and enhance patient health literacy competencies. Nevertheless, the authors are aware that adolescent patients find that additional components should be a part of the intervention as well.

### Data collection instruments and technologies

We used semi-structured interviews to explore the experiences and reflections on engaging with the health care system and health care providers for managing LBP. All adolescents were interviewed once, and the duration of the interviews were 20–40 min. Due to the COVID-19 pandemic, eight interviews were conducted through Microsoft Teams and two were conducted over the telephone. The interviewer (CLS) sat in an uninterrupted office through the interviews. The adolescents partook from a remote location of their choosing. The interviews were audio recorded via Dictaphones and field notes regarding communication behaviors (e.g., eye rolling, sighs, tone of voice or specific gestures) were also taken during the interview.

### Development and structure of the interview guide

The interview guide composed of 3 phases. In the first phase, the adolescents were encouraged to introduce themselves, their initial interest for participating and the clinical course of their own LBP. The second phase of the interview guide was based on key elements from health literacy literature (i.e., navigating the healthcare system, finding, and engaging with health care providers, and obtaining and processing information) and self-management literature (i.e., forming partnerships with health care providers, taking action and utilizing resources). At the end of the second phase, adolescents were asked to reflect on what they thought would be the most effective management strategies for their LBP and what they thought to be essential when managing adolescent LBP as a health care provider. In the third and final phase, adolescents was asked if they wished to speak of other matters related to their LBP which had not been covered during the interview and if they were surprised about some of the things discussed during the interview. The interview guide is available online as Additional file 1.

### Data processing

The audio recorded interviews were transcribed verbatim. NVivo 12 Pro for Windows were used during transcription and data analysis. To ensure anonymity among both the adolescents and the health care providers discussed in the interviews, highly specialized health care providers (e.g., orthopedic surgeon or rheumatologist) or treatment modalities (e.g., surgical procedure or pharmacological intervention) are presented as “specialist” and “specialized treatment” respectively.

### Data analysis

The 6-step model for thematic analysis described from 2006 by Braun and Clarke was used on the transcribed body of data [13]. As such, both a semantic (i.e., surface meaning, the “what”) and latent analysis (i.e., the search for deeper understanding, the “why”) of the data was undertaken [13, 14]. In the first step, CLS familiarized himself with the research material through listening and re-listing to the audio-based recordings and transcription of the material into written text [13]. During the second step, initial codes were generated by CLS. These codes were derived from the specific study aims. Specific themes and sub-themes were generated through the third step based on patterns identified within the initial codes [13]. The themes were then reviewed and validated from the research material during the fourth step. This process was preliminary undertaken by CLS and MSR and subsequently by the reaming authors. During the fifth step, the themes were defined and specified further before the finding were reported as part of step six [13]. The semantic analysis was descriptive in nature and was used to assess which circumstances made the adolescent seek care for their LBP, what type of treatment they received and which health literacy competencies they used while engaging with health care providers. The latent analysis was applied to investigate the deeper layers of care seeking behavior especially in terms of how the mechanisms and motivations behind the different self-management strategies would interact with each other. The thematic analysis approach is compatible with constructivism which was the epistemological standpoint within the study. As such, the study was conducted under the assumption that meaning and/or knowledge is produced through dialog between individuals [13, 15].

### Trustworthiness and transparency

Before undertaking the full thematic analysis, the interviewer (CLS) discussed the preliminary findings with a co-author (MSR) after analyzing six interviews. Based on this discussion, CLS subsequently analyzed the remaining four interviews. Afterwards, MSR went through 25% of the coding to verify the findings supporting the major themes and sub-themes. Due to logistics, the findings were discussed within the author group via email correspondence until consensus was reached. Saturation was reached after the 8th interview as no new themes or sub-themes arose during the remaining two interviews.

## Results

### Participant characteristics

Ten caucasian adolescents were recruited for the current study from November 17th, 2021, to June 29th, 2022. Four adolescents were recruited from primary care physiotherapy and medical clinics, 3 adolescents were recruited from sports clubs and 3 adolescents were recruited through social media posts. One semi-structured interview (median duration 34.5 min, range 20–41 min) was conducted with each of the ten adolescents. Characteristics of the participating adolescents are found in Table 1. Nine adolescents consulted a general practitioner during their course with LBP. The most common approach in general practice was a brief physical examination followed by referral for diagnostic imaging or physiotherapy. Eight adolescents consulted one or more physiotherapists during their course with the most common treatment modalities being exercises therapy and manual therapy. Five adolescents consulted a chiropractor with the most common treatment provided being spinal manipulation and massage.

**Table 1:** Characteristics of participating adolescents

Pseudonym	Age	Sex	Duration of LBP	LBP intensity*			Pain onset	Family history of LBP
				Avg	Worst	Current		
Kathrine	18	Female	1.5 years	6	8	4	Insidious	Yes
Sarah	17	Female	3 years	6	6	4	Insidious	No
Emma	18	Female	5 years	6	9	4	Traumatic	Yes
Hellen	16	Female	2–3 years	7	8	4	Insidious	Yes
Amanda	18	Female	2.5 years	5	8	0	Insidious	No
Beatrice	16	Female	3 years	0.5	3	0	Insidious	Yes
Diane	16	Female	2.5 years	6	7	4	Insidious	No
Tommy	16	Male	10 months	3	7	2	Insidious	No
Oliver	16	Male	9 months	0	2	0	Insidious	Yes
Ingrid	15	Female	2 years	7	9	4	Traumatic	No

* LBP intensity was measured on a 11-point numeric pain rating scale (0 = no pain & 10 = worst imaginable pain). LBP intensity was assessed as average LBP during last week, worst LBP during last week and current LBP

### Synthesis and interpretation

**Fig. 1:**
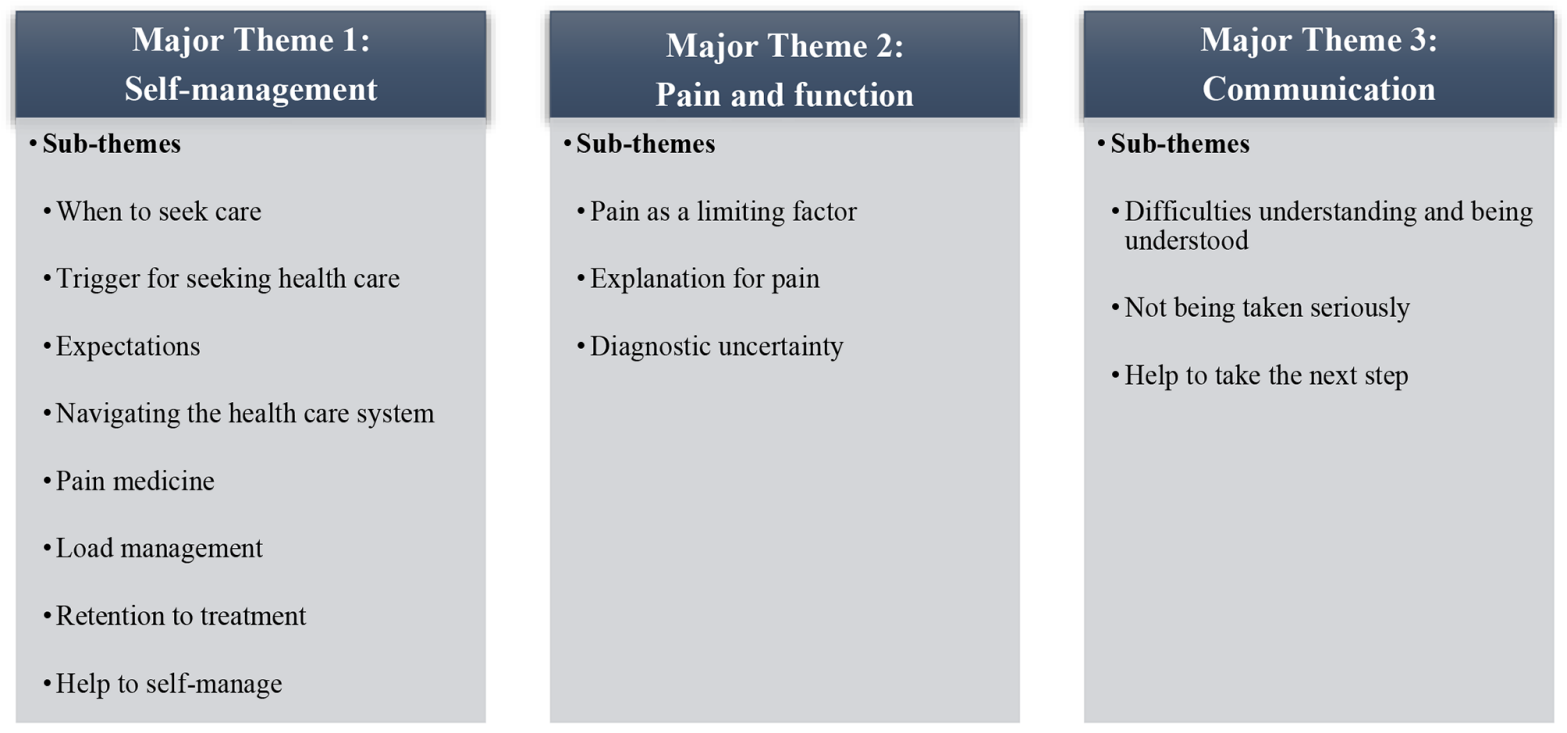
Overview of major themes and sub-themes

Three major themes emerged from the full thematic analysis: Self-management, Pain & Function, and Communication. Each major theme was comprised of 3–8 sub-themes which are outlined in Fig. 1. Both the major themes and sub-themes were based on the descriptive semantic part of the thematic analysis.

The findings from the descriptive semantic analysis can be found in Table 2 along with supporting sample quotes and the author’s interpretation. The latent part of the thematic analysis can be found below. In this section, we display how the findings of the study interact with the different core-skills of self-management as outlined by Lorig et al. [12].

**Table 2:** Interpretation of sub-themes with sample quotes derived from the semantic analysis

Sub-theme	Quotes	Interpretation
**Major theme 1: Self-management**		
Where to seek care	[How did you know to go to your general practitioner?]. *“… Those were the ones we could turn to and we had OK experiences with them previously*”. [Was it you or your parents that decided to contact the general practitioner?]. “*I think it was a mix. We had a good talk about it*.” – Beatrice, 16. [Was it you who decided to go to your general practitioner?]. “*I can’t really remember but my initial thought would be that it was my coach that recommended me and my parents to seek care from a general practitioner as it (the pain) had not improved during a full week.*” – Amanda, 18.	All adolescents decided to seek care in collaboration with their parents. Most adolescents chose to seek a given health care provider based on guidance from either parents or friends. Among those active in sport, many sought advice from their coach or friends from their sports-environment.
Trigger for seeking care	[When did you know it was the right time to see your general practitioner?]. “*Well, I said that I wanted to go to the general practitioner because it (the pain) had gotten so bad and my mom said that going to the general practitioner would be a good idea because she also thought that it (the pain) had gotten bad lately.*” – Hellen, 16. [What was your reason for seeking care with a physiotherapist again?]. “*That was because I bent down forward to pull my pants up and then I got a violent jab in the right side of my lower back and afterword I could not straighten myself out, I couldn’t move my arms up and I couldn’t walk, I couldn’t do anything.*” - Emma, 18.	Despite all adolescents being functionally limited by their LBP, the main trigger for seeking health care was continuous pain or a sudden increase in pain intensity.
Expectations	[What were your expectations beforehand (general practitioner)?]. “*Just to get referred on to a physiotherapist*” [Why did you decided to go to the doctor?]. “*That was actually my mom’s suggestion.*” – Kathrine, 18. [What was your expectation beforehand (general practitioner)?]. “*I was very optimistic that he would find out what was wrong so I could move on and not have pain anymore. I did not expect it to be a long process because I did not thing it would be this complicated. But he said that he could not figure out what was wrong so he didn’t do much and said that I should just keep taking pills and then he referred me to another doctor.*” – Hellen. 16.	The expectations before going to the general practitioner varied among the adolescents. Some had high expectations in terms of examination, diagnostics, and treatment while others only consulted to get referred to another health care provider
Navigating the healthcare system	[Were you ever in doubt about who to contact regarding your back pain?]. “*No, my general practitioner has always been our general practitioner even since I was a baby so my mom contacted him. We always contact him when something is up.*” – Hellen, 16. *“To me it has always been pretty much like, if you have problem then it is your general practitioner (that you go to) and then the general practitioner can often refer you to other places if they themselves can’t help you with it (the problem). I think I will get the most out of going to the general practitioner instead of for instance going to the internet because you do not get much out of that.”* Sarah, 17.	Some adolescents displayed sufficient health literacy skill as they knew how to navigate the different aspects of the Danish healthcare system. As such, many adolescents knew to consult their general practitioner initially due to their role as gatekeepers. The were also aware that the general practitioners were able to refer them one if needed.
Pain medicine	[Reflecting on own ability to self-manage]. “*Well, when I have to do something about it (the pain) myself then I just take those pills (pain medicine) but they do not really make any difference in terms of me getting better because they only dampen the pain.*” Hellen, 16. [Have you taken pain medicine for your back pain?]. “*Yes, during periods where it (the pain) was really bad*.” [What was your experience with that?]. “*It worked during practice or during competition where I had to perform. And then the pain was often worse afterwards because I had used my back too much*.” – Beatrice, 16.	For most adolescents, over-the-counter pain medicine was the first attempt to self-manage their LBP. Many used over-the-counter pain medicine repeatedly and for long periods of time despite limited or no effect. Those active in sports frequently used over-the-counter pain medicine to be able to complete training or competition.
Load-management	*“… I must remember to say that I did feel a little improvement in my back pain after I went to the physiotherapist. However, it got worse again when I began practicing more.*” – Beatrice, 16. “*I am on the specialized treatment for 3-months… I began to feel better during that period because I do not train at all… I was able to train full(y) on 6-months after I stopped the specialized treatment…3–4 months after that it started to go downhill again because it (the back) was not really ready”*. [How much of your progress do you think is due to the specialized treatment and who much did the break from training influence the pain?]. “*I think it was like 50/50*” – Diane, 16.	Only a few of the adolescents active in sport seemed to be aware that their pain could be related to their training volume. Further, most of the sports active adolescents were prescribed a load-management/load-reduction strategy however, it was often not described as such by the health professional nor interpreted as such by the adolescents. Even though all the sports active adolescents experienced improvement in pain and function during periods with reduced training volume (load-management) they point to the active components (such as exercise or medicine) as the primary contributors to improvement.
Retention to treatment	[Reflecting on seeking care with a health care provider who previously provided short term pain relief].*” I went back because I had seen some physiotherapists and they could not really do anything for me, and the acupuncturist had at least done something. So I went back in hope that she could help me and that it (the pain relief) would be long-term*.” – Diane, 16 [What was your reason for discontinuing your course at the chiropractor?]. “*Well, that was because I did not feel that it did anything good.*” – Tommy, 16.	One of the strongest drivers to retain or revisit treatment modalities and health care providers was if the treatment had proven effective previously. This was true even if the positive effect had only lasted for a few days or even a few minutes the first time. Although not essential for all the adolescents, some were more inclined to discontinue if the purpose of the treatment modality was not clear or if it did not make sense to them.
Help to self-manage	[Did you miss anything during the course of your treatment for back pain?]. “*I actually think that could be something like having a health professional that you were connected to and whom you had the possibility of calling or emailing and say*, *I am actually a little worried because it (the pain) is moving down in my legs*, *and then there would actually be someone who replied. To just know that you had a health professional that could answer your questions or help when you were insecure.*” – Amanda, 18. [What do you think is the best treatment for your back pain?]. “*To get across the finish line with these exercises. Also, I think it would be good to build up some muscles and activate them. However, if it (the pain) continues I might get a hold on a physiotherapist who can check if these are the right exercises.*” – Sarah, 17.	Most adolescents expressed a desire to self-manage their LBP however, many had a need for guidance and validation from a health care provider in order to be confident in self-managing.
**Major theme 2: Pain and function**		
Pain as a limiting factor	[What does it mean to you to experience pain?]. “*It has been irritating me for some time because I can’t do anything and that can get frustrating… For instance, I have trouble bending forward and I feel like an old woman. I feel it is a little bit early to feel old when you are only 16*”. [Do you feel hampered by you back pain in other activities that you like to do?]. “*Train. I feel limited all the time when I train. I can’t train like I did before, not even close.*” – Diane, 16. [What does it mean to you to experience pain?]. “*It is very substantial in my opinion… I am almost limited in everything I do. You can say that I can do everything, but everything also hurts… for instance, this Monday I had to write (to) my teacher to get a home assignment because I am not able to stay seated for so long during class. It is like, we have 45 min (of class) and then a 10-minute break, but I am not able to sit for 45 min. It is the same when I am at work… So, I am very limited during both school and work*.” – Amanda, 18.	All adolescents were functionally limited by their LBP. They often report pain to be problematic and hampering in relation to school, work, sports, and social activities.
Explanation for pain	[In your own words, what do you think is wrong with your back?]. “*I really don’t know what the problem is. I think maybe my back isn’t strong enough or that was what I thought in the beginning. But now when I have trained so much, and it still haven’t got better I don’t know what is wrong… It can also be a tense nerve. I have also been wondering if that could be the reason.*” Hellen, 16. [You talked to two physiotherapists and one of them said that your back needed to be straightened out?]. “*Yes, he thought that I was crocked so he moved my hips around and cracked and straightened (me) out and all sorts of stuff*.” – Diane, 16.	Many adolescents had a purely biomechanical explanation for their back pain. This explanation was either deduced by themselves, or more commonly based on the explanation given by a health care provider.
Diagnostic uncertainty	[Reflecting on underlying reason for back pain]. “*Some believe that as I have pain in my body, my muscles get tense in the back. So that means I am going around and being tense all the time. Some believe that anyway* (underlying mistrust).” – Kathrine, 18. [What treatment do you think would be the best for your back pain]. “*I think it would be to figure out why I have to go around and have so much pain because it can’t be because I got an injury so many years ago… So, I really just want to know what is wrong with me so I can take my precautions in order to get better.*” – Emma, 18.	Many adolescents expressed continuous frustration regrading not knowing the cause of their LBP. Although some were provided with an explanation for their pain, some adolescents questioned this or even dismissed it entirely.
**Major theme 3: Communication**		
Difficulties understanding and being understood.	“*I went to the physiotherapist with my mom. I have always liked to have her come along because physiotherapists and medical doctors in general they can say things where you fall a bit behind because you don’t understand it*.” – Amanda, 18. “*The help I need, it needs to be very through because it can be completely unmanageable to call other grown people and not be completely sure what to say. So very thorough help otherwise I think young people may just forfeit.*” – Sarah, 17.	Many adolescents were explicit about not being able to understand what health care providers told them about their LBP. Also, some adolescents expressed a concern of not being understood by the health care provider during consultation.
Not being taken seriously.	*“… (at the consultation waiting room) at some point I overheard that the older people are taken much more serious and even though their pain threshold may even be lower, and we (the younger once) have more pain, we don’t get taken serious in the same way (as the older). We are just children and should be in good health even though we are not.*” – Tommy, 16 [Did you miss anything during the course of your treatment for back pain?]. “*Communication and to be taken 100% seriously so that they believe in you*” [What should not be a part of the treatment for adolescent back pain?]. “*I think that would be people that give up easily and thinkWell, young people don’t know what real pain is and it is probably not as bad as she thinks.*” – Emma, 18.	Many adolescents felt they were not taken seriously in relation to their back pain. They especially felt that health care providers did not believe that they experience as much pain as they say they do.
Help to take the next step.	[What is the most important when treating young patients with back pain?]. “*From my point of view is being understood very important. To have someone understand that, at least for me, that you get a little bit desperate. You want people to help you and to understand you. So, I think it is very important to try to explain to young people who it is all connected instead of saying*, *I can’t help you*, because what can we use that for? *We cannot do anything with that. I would much rather that health professionals took a little extra time to explain why can’t help or what they think could help you.*” – Amanda, 18. “*I have had a physiotherapist who gave up on me… I got a bunch of exercises, 10 I think, and I come back one day and I say that it doesn’t work and that the pain keeps getting worse and that I can’t stand doing them (the exercises) where the physiotherapist replies that, he think it is best that we stop because he can’t do anything more for me as all he try to do makes it (the pain) worse*…” – Emma, 18.	Many adolescents felt that they at some point in their course with LBP reached a standstill because their current health care provider stated that they could not aid them further. Furthermore, many subsequently experienced that the health care provider did not help them initiate “the next step” or provide them with knowledge of what to do from that point forward in terms of managing their pain.

### Utilizing resources and making decisions

For most adolescents, over-the-counter pain medicine was a common and often first attempt to self-manage their LBP. Even though many used over-the-counter pain medicine repeatedly and for extended periods of time, the effect was often small or insufficient. Among sports active adolescents, over-the-counter pain medicine was frequently used to complete a training session or participate in competition. This may reflect a lack of knowledge about the appropriate use of over-the-counter pain medicine and insufficient awareness of alternative self-management strategies.

The main trigger for seeking healthcare was either pain or a sudden increase in pain intensity. Functional limitations were often experienced along with pain, although these limitations were not reported as the main trigger. Most adolescents decided to seek care in collaboration with their parents. Many adolescents sought care with a general practitioner, or another health care provider based on guidance from either parents or friends. Their expectations before going to the general practitioner varied. Some had high expectations in terms of extensive examination, diagnostics workup, and treatment while others only consulted to get referred to another health care provider.

Most adolescents were aware that the general practitioner may be consulted initially due to their role as gatekeepers to secondary healthcare and additional primary healthcare. However, at times when the general practitioner was not available (e.g., bank holidays), they were forced to explore alternative strategies. Emma, age 18, elaborated on such scenario:

[You chose to go to the emergency department, why was that?]. “*It was because it was a Sunday and I knew that I could not stand it (the pain) until I was able to get in touch with my general practitioner, so I had to figure out something and get some pain medicine or something*”. [Was it the right time to go to the emergency department you think?]. “*I think so. I had been waiting for a while and I had taken the pain medicine I already had*”.

This is one of numerous examples displaying adolescents’ ability to navigate the (Danish) healthcare system based on prior knowledge.

### Problem solving and taking action

For many adolescents, the overall purpose of seeking care was to be provided with strategies that could diminish their pain and subsequently lead to recovery of function. The desire among adolescents to self-manage their pain was frequently expressed during the interviews. Amanda, age 18, shared her thought on this matter during her interview:

[Did you miss anything during the course of your treatment for back pain?]. “*I actually think that could be something like having a health professional that you were connected to and whom you had the possibility of calling or emailing and say “I am actually a little worried because it (the pain) is moving down in my legs” and then there would actually be someone who replied. To just know that you had a health professional that could answer your questions or help when you were insecure*”.

Just like Amanda, many adolescents stated that they required help to self-manage their pain in the form of guidance from a health care provider, to feel fully confident in managing their own LBP. As such, none of the adolescents seemed to have any intention of being dependent on a specific health care provider nor a specific treatment. In summation, the adolescents consulted a health care provider for guidance and to get strategies to manage their LBP which they could experiment with on their own. When in doubt or when they had no success with the provided strategies, they would seek care again for either assurance that what they were doing was right or to get new strategies that they could try to manage their LBP.

### Barriers to self-management and forming partnerships

Sufficient and productive communication is essential for partnerships between adolescents and health care providers to be successful. However, most adolescents were explicit about experiencing difficulties understanding what health care providers told them, especially in relation to the cause of their LBP. The adolescents would often report being provided with an overwhelming amount of information which was made even harder to comprehend due to the use of long and difficult words. Also, adolescents expressed a concern of not being understood by the health care providers. This was often related to a sense of not knowing what to say and what information to give the health care providers.

An additional contributing factor for poor communication was when the adolescents did not feel they were being taken seriously by their health care provider. Diane, age 16, elaborated on her experience with consulting her general practitioner:

[Was there any health professional that you would say you had a poor relation with during your course with back pain?]. “*My general practitioner. I do not feel that it was the right way to deal with it. When you come several times and says that your back hurts it is not without reason… There is a reason that I keep coming and say it hurts and that it does not get better… He did not really understand that… it is like, just because you are a child then the pain is not so bad*”.

Like Diane, many adolescents had the feeling that their health care provider would express scepticism towards their pain intensity. In extreme cases, adolescents felt that the health care provider did not believe that the pain was as intense as the adolescents described it. As such, poor communication and distrust could hamper a mutually beneficial partnership between adolescents with LBP and health care providers. For some adolescents, these barriers lead to feeling abandoned as the adolescent did not know how to move forward in terms of managing their LBP. Many adolescents felt that they at some point in their course with LBP reached a standstill as their current health care provider were not able to help them further. Consequently, some adolescents felt lost as they were not provided with help to take the next step in terms of treatment modalities or other guidance to seek other health care providers.

## Discussion

The findings from this study suggest adolescents consulting health care providers for LBP experience multiple barriers for establishing meaningful and productive partnerships to be able to self-manage their pain. The adolescents sought care due to pain or intensified pain after an initial failed attempt to self-manage their pain. They collaborated with their parents and peers prior to consulting a health care provider especially if they themselves had limited or no prior experience with the healthcare system. These findings suggest that adolescents with LBP can use the resources available to them (i.e., support and advice from parents and peers) and use these resources to take action (i.e., seek care). This is important as utilizing resources and taking action are both core-skills in self-management [12].

Most adolescents had been given a solely biomechanical explanation for their pain. This was despite the fact that all the adolescents were functionally limited which in some lead to a sensation of “*feeling old*”, “*irritated*” or even “*frustrated*”. As such, many of the adolescents showed signs of being affected on a psychosocial level which is also known to influence LBP in adolescents [16]. These findings are in line with a resent scoping review in which the authors found that interventions in previous non-surgical and non-pharmacological experimental studies in adolescents with recurrent or persistent LBP were based on outdated biomechanical models for persistent LBP [16] In some cases, adolescents found the biomechanical explanation to be unsatisfactory which in turn could lead to the feeling of diagnostic uncertainty. Even though many of the active adolescents were provided with a load management strategy (i.e., reduction in training volume) they would most often point to an active component (e.g., specific exercises or manual therapy) to be the main contributor to their pain reduction. This underlines the importance of providing accurate information to avoid misbeliefs regarding mechanisms for pain and pain relief.

Most adolescents expressed a desire to self-manage their LBP, but many expressed a need for guidance and validation from a health care provider to feel confident in self-managing their pain. Although self-management to some extent implies that patients should manage their illness or conditions, this does not mean that patients should be managing their pain on their own. Rather, patients and health care providers should form partnerships in which patients expand their skills in providing accurate and precise information about their pain experience for the health care provider to act on [12]. In this regard, establishing a strong therapeutic alliance or partnership with health care providers has previously been described as a central component in managing adolescent LBP [12]. Poor communication and the feeling of not being taken seriously was found to be a significant barrier for establishing a partnership between the adolescents and health care provider which in turn would hamper the adolescents in self-managing their pain in collaboration with a health care provider.

### Comparison with existing literature

Prior to seeking care many adolescents used over-the-counter pain medicine as a first step to self-manage their pain although this approach had short lasting or no effect on their pain. Over-the-counter pain medicine was used frequently and for long periods among the sport active adolescents which aligns with the findings in a recent review on the use of over-the-counter pain medicine among adolescents [17]. With problem solving being one of the 5 core-skills of self-management [12] our findings indicate that care-seeking adolescents with LBP need alternative strategies when trying to self-management.

This is important as we further uncovered a general desire among the adolescents to self-manage their condition with guidance from a health care provider. However, our findings indicate that poor communication and the feeling of not being taken seriously among the adolescents may severely hamper the possibility to form productive partnerships. The adolescents point to this as especially problematic in situations where their treatment courses are discontinued without being advised on a possible next step. In these situations, the adolescents felt that the health care providers gave up on them despite their young age. This reaction from the adolescents indicate that they did not have the required knowledge to make a decision for their future treatment course on their own. This is problematic as both being able to make a decision and forming partnerships with health care providers are vital components when self-managing a condition [12].

Communication barriers between adolescent patients and health care providers have previously been highlighted however, here the problem often relates to the adolescents’ underdeveloped abilities to communicate, make informed decisions, and assess potential threats [18]. Nevertheless, this standpoint is somewhat fixed and offers no solution. In a recent viewpoint by Pate and colleagues, the authors highlight the importance of not assessing adolescents LBP patients as adult patients [19]. The authors especially encourage health care providers to prioritize listening to the adolescents’ narrative and to ask open-ended questions such as “Tell me about why you have come to see me today?” [19]. With health care providers recognizing the need for an individually tailored approach to assessing adolescents with LBP, communication between the two parties is likely to improve [18, 19].

### Clinical implications

Acknowledging the importance of communication when managing LBP among care-seeking adolescents is vital to foster a mutually beneficial partnership between the health care provider and the adolescent. This may be especially true to prevent that some adolescents feel they are not being taken serious or in worst case experience distrust from their health care provider during the consultation. Also, it is important to recognizing adolescent LBP as more than a purely biomechanical conditions. The adolescent informants in the current study expressed both frustration and irritation towards their pain experience indicating the presence of a psychological component. This is in line with current evidence regarding the experience of adolescent LBP [20].

#### Implications for future research

Based on the findings from the current study, there seems to be a rational to further explore how health care providers can support adolescents in self-managing their LBP. Most of the adolescents in the current study had an explicit desire to self- manage their LBP but they also expressed a need for continuous sparring and guidance from a health care provider. As such, it may be possible for primary care general practitioners or physiotherapists to assume the roles of health care consultants when supporting care-seeking adolescents with LBP. This approach may in addition facilitate the adolescent to become an active part of the management process. Furthermore, the adolescents from the current study displayed sufficient abilities to navigate the Danish healthcare system especially in collaboration with family members (i.e., parents). As utilizing the resources among the parents positively influenced the process with navigating the healthcare system, future studies should explore how and if parental resources may positively influence the self-management process of care-seeking adolescents with LBP.

### Strengths and limitations

This study was initiated during the COVID-19 pandemic and therefore all interviews were undertaken online through Microsoft Teams or through telephone calls. This approach made it possible to interview adolescents from all of Denmark as transportation or COVID-19 was not a barrier for participating. However, the online format may have influenced the interview process due to the lack of physical presence between the adolescent and the interviewer. A counter argument would be that the online format gave the adolescents the possibility to participate from a remote location of their choosing, without the pressure of visiting a university or having to invite a foreign person (the interviewer) into their home. Instead, the adolescents could interact with interviewer at a distance through the computer or phone. One limitation is that two interviews were conducted over the phone. This approach was used as there was technical issues with using Microsoft Teams. From this alternative approach it was not possible to take field notes as described in the section of data collection instruments and technologies. As such, the conducting the two interviews in question over the phone was the best possible options at the time. As the duration of the interviews in general were short, this may pose and additional limitation. However, with a limited number of interview studies in this specific research area, is it difficult to estimate how long and interview should last. In a previous study by Lauridsen and colleagues, the authors recruited children between 9 and 12 years of age from two public schools in Denmark and interviews them about what they considered to be important consequences of having LBP [21]. In this study, the interviews lasted between 15 and 30 min [21]. As the informants in that study was younger than the once in the current study, the discrepancy between the duration of the interviews may reflect the age of the informants and the content of the interview guide. Lastly, the small number of informants may be considered a limitation of the current study. However, as previously described, data saturation was reached after the 8th interview as no new themes or sub-themes arose during the remaining two interviews. A similar approach in was used in the study by Lauridsen and colleagues [21]. In relation, based on the semantic and latent analysis we believe that the 10 adolescent informants were an adequate sample to answer the present research question. A major strength is the inclusion of adolescents who had recent contact with a health care provider and functional limitations as this made it possible for us to gain insight into the level of health literacy and self-management skills among care-seeking adolescents with LBP.

## Conclusion

Our findings indicate that adolescents with LBP from the current study display various health literacy competencies which partly enables them to navigate the healthcare system in collaboration with their parents in order to seek care. However, the adolescents lacked alternative self-management strategies as initial attempts to self-manage with over-the-counter pain medicine would often be ineffective. Most adolescents were explicit about their desire to self-manage their LBP, but they needed guidance from a health care provider in order to be confident in self-managing their pain. One of the most common barriers for engaging in a partnership with a health care provider was poor communication and the feeling of not being taken seriously. Health care providers should strive to tailor the assessment of adolescents with LBP to optimize communication. Further, health care providers should assist adolescents self-manage their pain and provide guidance when needed.

### Electronic supplementary material

Below is the link to the electronic supplementary material.

Supplementary Material 1

## Data Availability

We did not obtain consent to share audio or transcribed data from the current study. As such, data cannot be shared.

## Abbreviations

LBP: Low back pain
