# Supplementary material for: Competences to self-manage low back pain among care-seeking adolescents from general practice - a qualitative study
Source: BMC Prim Care. 2023 Nov 29;24:252. doi: 10.1186/s12875-023-02212-4 (PMC10685513; doi:10.1186/s12875-023-02212-4)
Supplement: Supplementary file 1 — Supplementary Material 1 [file 12875_2023_2212_MOESM1_ESM.docx]

**Additional file 1: Interview guide**

| Phases | Domaine | Sub-domain | Questions | Purpose of the questions |
| --- | --- | --- | --- | --- |
| Phase 1. | Introduction | Introduction | I would like to ask if you would start out by telling me a little about yourself and what made you interested in participating in this project. | This question is used to get the respondents to talk about themselves and getting comfortable with the interview setting. This also provides a possibility to steer the conversation in the direction of the project. |
|  | Experiences | The clinical course | Tell me about your course with low back pain. Start with the beginning. | This question is used to gain insight into the clinical course of the respondents. This also provide the opportunity to investigate how the pain originated (i.e., traumatic or insidious). |
|  |  | Pain | What does it mean to you to have, and experience, pain. | This question is used to gain an understanding of which meaning the respondents have assign to the word “pain” and the experience of having pain. |
|  |  | Previous treatment strategies | What kind of treatments have you received for your back pain? | This question is used to supplement the previous question. This question also investigates if the respondents were offered or received different treatments from different providers. |
|  |  | Previous treatment strategies | Which treatments have you received that reduced your back pain. | This question is used to gain insight into which treatments led to an experience of pain relief among the respondents. |
|  |  | Previous treatment strategies | Which treatments have you received that increased your back pain. | This question is used to gain insight into which treatments led to an experience of increase in pain among the respondents. |
|  | Self-management Skills | Taking action | Why did you choose to go to a health care provider?  How did you (or your parents) know that it was the right time to seek care? | The purpose of this question is to uncover the exact reason for seeking care among the respondents.  The subsequent question is used to gain insight into why the respondents believed the time was right to seek care. |
|  | Self-management Skills | Taking action | Looking back would you have done things differently regarding the time of your consultation? Would you have consulted sooner or later than you did?  If so, why is that? | This question is used to assess if the respondents would have done things differently in terms of seeking care and if so, why they would have done things differently. |
|  | Health literacy competencies | Care pathways | What considerations did you have regarding who to contact?  Were you ever in doubt about who to contact?  If so, why was that? | This question is used to gain insight into the considerations the respondents had prior to seeking care with a health care provider.  The subsequent question will help uncover if the respondents was ever in doubt about who to contact and if so, why they were in doubt. |
|  | Health literacy competencies | Health-related self-efficacy | If you think about where you are today, to what extent do you feel able to manage your back pain?  If you think of a 0-10 scale with 0 equals that you do not feel able at all to mange your back pain and 10 equals that you feel completely able to manage your back pain, which number do you then think best reflect how able you feel in managing you back pain?  Try to say a few words about why you chose that number. | This initial question is used as a proxy to assess if the respondents believe they are able to manage their pain (i.e., pain self-efficacy). The subsequent question is used to uncover the underlying considerations for why the respondents chose the number that they did.  This provides the opportunity to assess which strengths and weaknesses the respondents believe will impact their ability to manage their back pain. |
|  | Health literacy competencies | Partnership | Which health care provider did you feel especially good working with during your course of back pain?  Why do you think you felt especially good working with that person? | This question is used to uncover if the respondents have had any good relationships with a health care provider and also make the respondents reflect on why they believe this relationship was especially good. |
|  | Health literacy competencies | Partnership | Which health care provider did you feel especially awful working with during your course with back pain?  Why do you think you felt especially awful working with that person? | This question is used to uncover if the respondents have had any very bad relationships with a health care provider and also make the respondents reflect on why they believe this relationship was especially awful. |
|  | Health literacy competencies | Utilization of resources | Which kind of treatment did you expect to receive when you contacted your health care provider?  Did the treatment you received match your prior expectations?  Why do you think that? | This question is used to gain insight into any prior expectations the respondents had regarding a certain treatment. This is especially important if the respondents received another treatment than expected. As such, the question is followed by two subsequent questions with the last one assessing if the respondents experienced a discrepancy between their expectations and what they actually received. |
| Phase 2. | Visions | Treatment content | I would like you to describe what you think would be the best treatment for your back pain. | The question is used to assess what the respondents themselves believe will ease their back pain. |
|  |  |  | What do you think is the most important to include in treatment aimed at young individuals with back pain? | This question is used to assess what the respondents believe to be essential to include in a treatment course aimed at young individuals with back pain. |
|  |  |  | I would like you to state if you think there was something missing from the treatment you have received for your back pain. | This question is used to make the respondents reflect on which elements (if any) they lacked from their own treatment course. |
|  |  |  | In your opinion what should definitely not be a part of the treatment for young individuals with back pain? | This question is used to assess what the respondents believe should definitely not be a part of a treatment course aimed at young individuals with back pain. |
| Phase 3. | Ending the interview | Closing remarks and reflections | Is there anything that you would like to add before we conclude the interview?  Did any of the things we discussed surprise you?  Thank you very much for your participation. | This question is used to give the respondents space to contribute with final remarks and reflection and also to round up the interview. |

**Legend**: The interview guide has been translated from Danish into English after the interviews were conducted.
